# Comparative Analysis of Generative Artificial Intelligence Systems in Solving Clinical Pharmacy Problems: Mixed Methods Study

**DOI:** 10.2196/76128

**Published:** 2025-07-24

**Authors:** Lulu Li, Pengqiang Du, Xiaojing Huang, Hongwei Zhao, Ming Ni, Meng Yan, Aifeng Wang

**Affiliations:** 1 Department of Pharmacy, Fuwai Central China Cardiovascular Hospital, 1 Fuwai Road, Zhengdong New District, Zhengzhou, China, 86 18538298379

**Keywords:** artificial intelligence, DeepSeek-R1, clinical pharmacy, comparative analysis, generative AI

## Abstract

**Background:**

Generative artificial intelligence (AI) systems are increasingly deployed in clinical pharmacy; yet, systematic evaluation of their efficacy, limitations, and risks across diverse practice scenarios remains limited.

**Objective:**

This study aims to quantitatively evaluate and compare the performance of 8 mainstream generative AI systems across 4 core clinical pharmacy scenarios—medication consultation, medication education, prescription review, and case analysis with pharmaceutical care—using a multidimensional framework.

**Methods:**

Forty-eight clinically validated questions were selected via stratified sampling from real-world sources (eg, hospital consultations, clinical case banks, and national pharmacist training databases). Three researchers simultaneously tested 8 different generative AI systems (ERNIE Bot, Doubao, Kimi, Qwen, GPT-4o, Gemini-1.5-Pro, Claude-3.5-Sonnet, and DeepSeek-R1) using standardized prompts within a single day (February 20, 2025). A double-blind scoring design was used, with 6 experienced clinical pharmacists (≥5 years experience) evaluating the AI responses across 6 dimensions: accuracy, rigor, applicability, logical coherence, conciseness, and universality, scored 0‐10 per predefined criteria (eg, −3 for inaccuracy and −2 for incomplete rigor). Statistical analysis used one-way ANOVA with Tukey Honestly Significant Difference (HSD) post hoc testing and intraclass correlation coefficients (ICC) for interrater reliability (2-way random model). Qualitative thematic analysis identified recurrent errors and limitations.

**Results:**

DeepSeek-R1 (DeepSeek) achieved the highest overall performance (mean composite score: medication consultation 9.4, SD 1.0; case analysis 9.3, SD 1.0), significantly outperforming others in complex tasks (*P*<.05). Critical limitations were observed across models, including high-risk decision errors—75% omitted critical contraindications (eg, ethambutol in optic neuritis) and a lack of localization—90% erroneously recommended macrolides for drug-resistant *Mycoplasma pneumoniae* (China’s high-resistance setting), while only DeepSeek-R1 aligned with updated American Academy of Pediatrics (AAP) guidelines for pediatric doxycycline. Complex reasoning deficits: only Claude-3.5-Sonnet detected a gender-diagnosis contradiction (prostatic hyperplasia in female); no model identified diazepam’s 7-day prescription limit. Interrater consistency was lowest for conciseness in case analysis (ICC=0.70), reflecting evaluator disagreement on complex outputs. ERNIE Bot (Baidu) consistently underperformed (case analysis: 6.8, SD 1.5; *P*<.001 vs DeepSeek-R1).

**Conclusions:**

While generative AI shows promise as a pharmacist assistance tool, significant limitations—including high-risk errors (eg, contraindication omissions), inadequate localization, and complex reasoning gaps—preclude autonomous clinical decision-making. Performance stratification highlights DeepSeek-R1’s current advantage, but all systems require optimization in dynamic knowledge updating, complex scenario reasoning, and output interpretability. Future deployment must prioritize human oversight (human-AI co-review), ethical safeguards, and continuous evaluation frameworks.

## Introduction

With the breakthrough development of generative artificial intelligence (AI) technology, the health care field is experiencing a significant transformation, with AI-driven pharmaceutical practice at the forefront of this evolution [1]. Pharmaceutical intelligence has the potential to transform pharmaceutical practice by addressing the complexity of drug data, evolving health care needs, and technological advancements. Global research indicates that AI demonstrates transregional universality in enhancing the efficiency of drug information retrieval [2]. Clinical studies indicate that such systems demonstrate significant advantages in efficient drug information retrieval and exhibit a certain degree of accuracy and specificity in predicting drug interactions [3]. However, recent systematic reviews point out that current AI chatbots primarily face challenges of “generating inaccurate or fabricated content” and “lower accuracy in answering questions” [4,5]. This gap between technological potential and practical application [6,7] highlights the urgency of establishing scientific evaluation systems to identify high-quality generative AI systems.

Despite some existing research beginning to conduct clinical application assessments of generative AI dialogue systems, these efforts are largely limited to testing individual models on single tasks [8-10], lacking horizontal comparative analysis across multiple dialogue models and validation of continuous decision chains in real clinical scenarios.

This research innovatively constructs a 6-dimensional evaluation system, conducting systematic assessment and comparative analysis of 4 types of clinical pharmacy practice scenarios: medication consultation, medication education, prescription review, and case analysis with pharmaceutical care. The study sample encompasses 8 representative mainstream dialogue-based AI platforms from both domestic and international origins: ERNIE Bot (version 4.0; Baidu; Release Date: October 17, 2023), Doubao (version: Pro; ByteDance; Release Date: May 15, 2024), Kimi (version: V1.1; Beijing Moonshot Technology Co., Ltd.; Release Date: November 16, 2023), Qwen (version: long; Alibaba Cloud; Release Date: May 21, 2024), GPT (version: 4o; OpenAI; Release Date: May 14, 2024), Gemini (version: 1.5-Pro; Google DeepMind; Release Date: February 14, 2024), Claude (version: 3.5-Sonnet; Anthropic; Release Date: June 21, 202), and DeepSeek (version: R1; Hangzhou DeepSeek Artificial Intelligence Basic Technology Research Co, Ltd; Release Date: January 20, 2025). Through designing parallel tests in realistic clinical settings and using a modified Delphi method for double-blind evaluation, we quantitatively analyzed and descriptively evaluated the capabilities of these 8 generative AI dialogue systems in addressing clinical pharmacy problems across 6 dimensions: accuracy, rigor, applicability, logical coherence, conciseness, and universality. The research findings will provide empirical evidence for optimizing the application of generative AI systems in clinical pharmacy and offer valuable reference for constructing AI-assisted decision-making systems that conform to medical ethics.

## Methods

### Research Design

We collected 48 common questions from 4 categories of clinical pharmacy work scenarios:

1. Medication consultation questions (n=20) content covered 10 aspects, with 2 questions per aspect: drug indications (efficacy), administration methods, dosage, medication precautions, drug interactions, storage methods, identification and management of adverse drug reactions, special dosage form usage guidance, medication use in special populations, and disease prevention.
2. Medication education questions (n=10) content primarily covered medication use for patients with chronic diseases and special populations.
3. Prescription audit questions (n=10) content encompassed inappropriate treatment regimens, inappropriate usage and dosage, inappropriate combination therapy, inappropriate drug selection, inappropriate administration routes, contraindications, inappropriate treatment duration, and inappropriate clinical diagnosis. The task required assuming the role of a pharmacist to identify prescription errors using relevant pharmacotherapeutic knowledge and the latest clinical guidelines.
4. Case analysis and pharmaceutical care questions (n=8) content included common chronic disease cases such as coronary heart disease, hypertension, type 2 diabetes, asthma, chronic obstructive pulmonary disease, gout, lung cancer, and so on. The task required assuming the role of a pharmacist to analyze pharmacotherapy plans based on patient information (basic information, reason for visit, present illness history, past medical history, medication history, family history, allergy history, adverse reaction history, unhealthy habits, diagnosis, current medication records, and auxiliary examination results), and to develop a pharmaceutical care plan addressing 4 aspects: indications, effectiveness, safety, and adherence.

The study used a standardized experimental design, with 3 researchers using identical “inquiry prompts” to question 8 generative AI dialogue systems during the same time period. All models were tested using their publicly available versions as of February 20, 2025; the results reflect a performance snapshot restricted to this timepoint. Each chatbot received 48 inquiry prompts, generating a total of 384 independent response samples. The evaluation was conducted by 6 clinical pharmacists who had successfully obtained clinical pharmacist training certificates after standardized training and had more than 5 years of clinical pharmacy work experience. The evaluation encompassed six dimensions: (1) accuracy, (2) rigor, (3) applicability, (4) logical coherence, (5) conciseness, and (6) universality. These clinical pharmacists’ professional domains covered all disease types relevant to the questions. A double-blind scoring mechanism was implemented for independent evaluation to avoid subjective bias and ensure objectivity and fairness in the assessment process.

Standardized prompting instructions: all questions were input to the model using a standardized format. The core instruction template was to act in the role of a clinical pharmacist. Based on the latest clinical guidelines and evidence-based principles, answer the following question (Specific question description). For prescription review tasks, the following emphasis was added: determine whether this prescription contains errors and provide your rationale. For case analysis and pharmaceutical care tasks, the following emphasis was added: analyze the pharmacotherapy plan for this case and develop a pharmaceutical care plan addressing the following 4 aspects “Indication, Efficacy, Safety, and Adherence.” The complete list of all 48 standardized prompting instructions used in this study is provided in Multimedia Appendix 1.

### Data Sources

Questions were collected using stratified sampling, drawing from the following sources: common medication inquiries at a medication consultation clinic in a large Grade A Class 3 hospital in China, real clinical cases, the theoretical assessment question banks for standardized clinical pharmacist training programs of the Chinese Medical Association (CMA) and the Chinese Hospital Association (CHA), the China Medication Therapy Management (MTM) pharmacist training program, and the China Pharmacist Skills Competition. Specific steps are as follows:

Question bank construction: questions from the 5 sources above were categorized into 4 scenarios: medication consultation, medication education, prescription review, and case analysis and pharmaceutical care.

Stratified sampling:

- Medication consultation (20 questions): ensured coverage of the 10 aspects mentioned in the Abstract (medication indications, administration methods, dosage, precautions, drug-drug interactions, storage methods, identification and management of adverse drug reactions, guidance on special dosage forms, medication use in special populations, and disease prevention). Two questions were randomly selected from each aspect.
- Medication education (10 questions): randomly selected from 2 subcategories, medication use for patients with chronic disease (6 questions), and medication use for special populations (4 questions).
- Prescription review (10 questions): ensured coverage of the 8 types of inappropriate prescriptions mentioned in the Abstract (inappropriate therapeutic regimen, inappropriate dosage and administration, inappropriate combination therapy, inappropriate drug selection, inappropriate route of administration, presence of contraindications, inappropriate duration of therapy, and inconsistency with clinical diagnosis). Prescription cases containing typical or high-risk errors were prioritized.
- Case analysis and pharmaceutical care (8 questions): 8 representative cases were randomly selected from the question bank covering common chronic diseases (eg, coronary heart disease, hypertension, type 2 diabetes, asthma, chronic obstructive pulmonary disease, gout, and lung cancer).

Question screening: the initially screened questions were independently reviewed by 2 senior clinical pharmacists. This process ensured the questions aligned with the assessment objectives, possessed clinical relevance, were clear and unambiguous, and excluded duplicate or outdated items. Ultimately, 48 questions were finalized for inclusion in the study.

### Evaluation Methods

Scoring standards were constructed based on evidence-based medical references, including drug package inserts, the latest clinical guidelines, and the Micromedex database. Clinical pharmacists conducted quantitative assessments of response contents according to standard answers and scoring criteria, with accuracy levels represented on a 0‐ to 10-point scale. Unified scoring rules were established, with each question having a maximum score of 10 points, with deductions as follows:

1. Accuracy: 3 points deducted for not directly addressing the question or not providing an accurate answer;
2. Rigor: 2 points deducted for incomplete answers;
3. Applicability: 2 points deducted for failing to provide individualized recommendations based on patient-specific conditions;
4. Logical coherence: 1 point deducted for unclear reasoning or disorganized logic;
5. Conciseness: 1 point deducted for verbose language;
6. Universality: 1 point deducted for overly technical terminology lacking general applicability;
7. Comprehensive answers without deduction criteria: 0 points deducted.

Evaluators were required to select at least one scoring option during the assessment process.

### Statistical Analysis

One-way ANOVA was used to compare score differences among the 8 generative AI dialogue systems across 4 task categories (medication consultation, medication education, prescription review, and case analysis with pharmaceutical care). Data satisfied the assumptions of normality through Shapiro–Wilk tests (*P*>.05) and homogeneity of variance through Levene tests (*P*>.05), followed by Tukey Honestly Significant Difference (HSD) multiple comparison analysis to identify significant differences. Interrater consistency was calculated using the intraclass correlation coefficient (ICC) through a 2-way random effects model, with ICC>0.75 indicating good consistency.

### Qualitative Analysis Process

Following quantitative scoring, the research team (3 investigators) conducted a systematic content review of all 384 AI-generated responses to identify recurrent strengths, typical error patterns, critical limitations, and notable variations across models. The review process followed the steps mentioned below.

Initial screening: investigators independently reviewed all responses, documenting exemplar responses that demonstrated exceptional performance or significant deficiencies in accuracy, rigor, applicability, logical coherence, conciseness, or generalizability.

Theme identification: investigators consolidated preliminary observations and identified recurring themes through discussion (eg, “Errors in Special Device Instructions,” “Lack of Localized Medication Recommendations,” “Failure to Identify Critical Contraindications,” “Overly Technical or Jargon-Rich or Verbose Language,” and “Ignoring Conflicting Gender-Specific Diagnostic Criteria”).

Case selection: for each identified theme, representative response examples demonstrating the issue or strength were selected across different models. Priority was given to cases where quantitative assessment revealed performance variations and where the response content clearly illustrated the qualitative concern.

Content extraction and verification: key content was abstracted from selected cases to ensure descriptions accurately reflected the original response meaning (with critical verbatim excerpts cited where necessary). Final qualitative cases and their analyses were confirmed by consensus within the research team.

**Figure 1.**
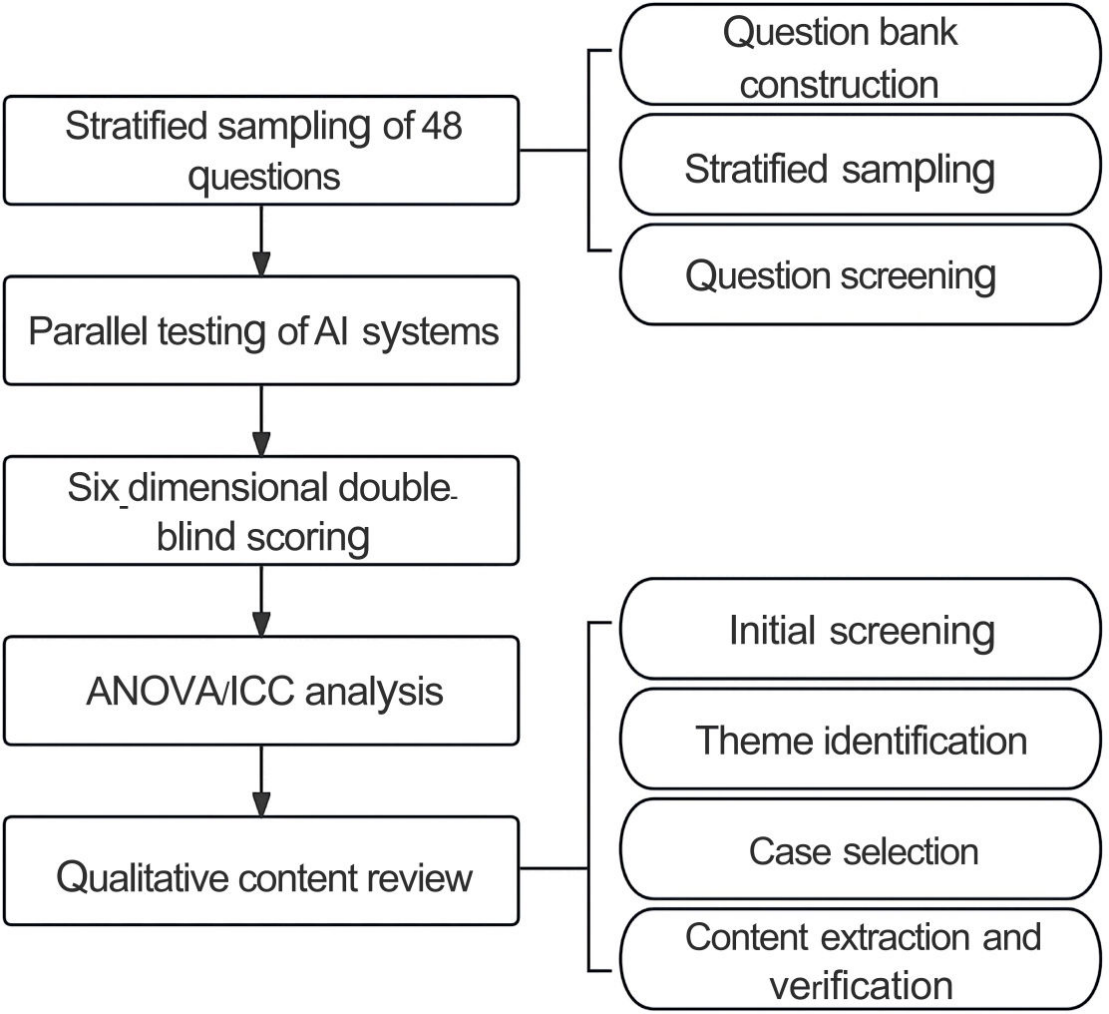
Research methodology flow diagram. AI: artificial intelligence; ICC: intraclass correlation coefficient.

These qualitative findings supplement the quantitative results, providing deeper insight into model performance variations and potential risks across different clinical scenarios. They are reported and analyzed in detail in the Discussion section. The methodology flowchart is shown in Figure 1.

### Ethical Considerations

This study constitutes a noninterventional evaluation of outputs from publicly accessible generative AI systems. It does not involve direct participation, intervention, or interaction with human participants (patients or healthy volunteers).

According to the policy of the Ethics Committee of Fuwai Central China Cardiovascular Hospital, this type of research (performance evaluation of publicly available AI systems using deidentified question banks and simulated scenarios) is qualified for exemption from formal ethics review application. The study design strictly followed the principles of the Declaration of Helsinki concerning nonbiomedical research.

This study exclusively used rigorously deidentified question texts as input stimuli for the AI systems. It did not involve the use, storage, or analysis of any raw patient data containing personally identifiable information. Therefore, additional informed consent for this secondary analysis was not required.

All question texts input into the AI systems were based on simulated scenarios or deidentified, generic inquiries, containing no real, personally identifiable patient information. The AI-generated output texts produced during the study contained only clinical pharmacy knowledge-related responses and similarly did not involve any private personal data. All research data (question bank, AI responses, and scoring sheets) were encrypted during storage and transmission and were accessible only to authorized researchers.

The 6 clinical pharmacists participating in the assessment received market-standard honoraria commensurate with their professional contribution.

**Figure 2.**
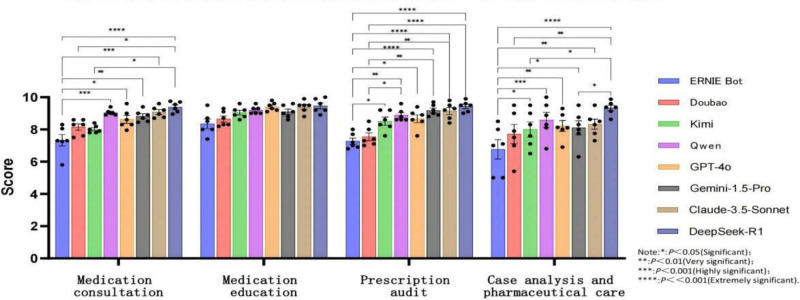
Score distribution and differential analysis of artificial intelligence platforms across 4 pharmaceutical tasks (box plot). (Note: *P<.05: significant difference, **P<.01: highly significant difference, ***P<.001: extremely significant difference, ****P<<.001: most significant difference).

**Figure 3.**
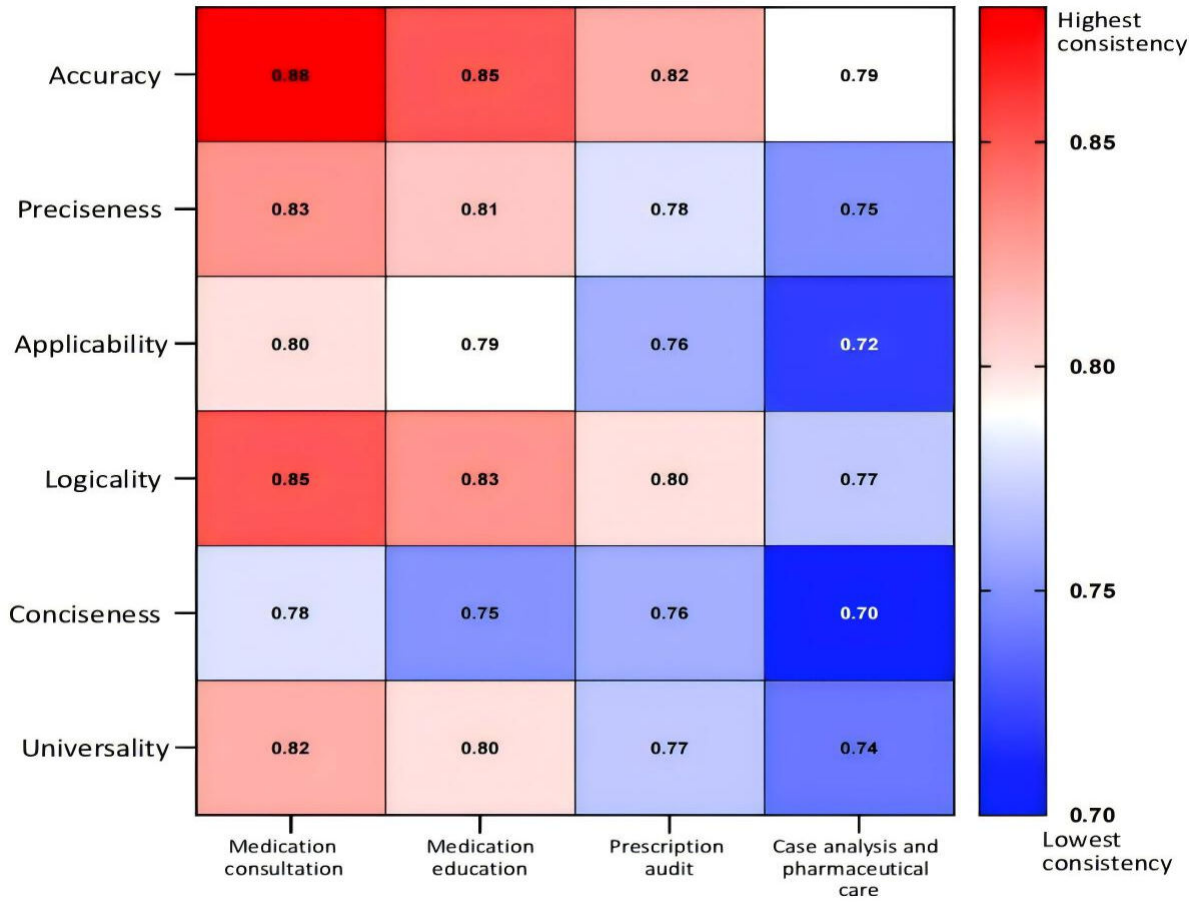
Rater consistency heat map (heat map of intraclass correlation coefficients).

This study did not use any images containing personally identifiable information (eg, faces, unique physical characteristics, and personal details). All results presentation figures (eg, Figures 1-3) were generated based on aggregated statistical data and anonymized ICC values, posing no risk of individual identity disclosure.

## Results

### Quantitative Evaluation of 8 Generative AI Dialogue Systems in Clinical Pharmacy Applications

#### Descriptive Statistics

DeepSeek-R1 demonstrated the strongest comprehensive capabilities, with particularly significant advantages in complex tasks (such as case analysis and pharmaceutical care). Qwen, GPT-4o, Claude-3.5-Sonnet, and Gemini-1.5-Pro performed exceptionally in certain tasks but were overall inferior to DeepSeek-R1. Doubao and Kimi showed inconsistent performance, while ERNIE Bot performed the poorest, indicating a need for targeted optimization. The SDs for scores in case analysis and pharmaceutical care tasks were relatively large, suggesting poorer consistency among clinical pharmacists when evaluating generative AI dialogue systems’ performance in handling these complex issues (see Table 1).

**Table 1.** Scores of 8 generative artificial intelligence (AI) dialogue systems across 4 problem types.

AI platforms	Medication consultation (n=20), mean (SD)	Medication education (n=10), mean (SD)	Prescription audit (n=10), mean (SD)	Case analysis and pharmaceutical care (n=8), mean (SD)
ERNIE Bot	7.3 (1.2)	8.4 (0.9)	7.3 (1.0)	6.8 (1.5)
Doubao	8.1 (1.1)	8.7 (1.3)	7.6 (1.2)	7.7 (1.7)
Kimi	8.0 (1.0)	9.1 (1.0)	8.5 (1.4)	8.0 (1.6)
Qwen	9.0 (0.8)	9.2 (0.7)	8.9 (0.9)	8.6 (1.2)
GPT-4o	8.6 (1.0)	9.4 (1.1)	8.7 (1.3)	8.2 (1.4)
Gemini-1.5-Pro	8.8 (1.1)	9.1 (1.0)	9.2 (1.1)	8.1 (1.3)
Claude-3.5-Sonnet	9.1 (1.0)	9.4 (0.8)	9.2 (0.9)	8.3 (1.5)
DeepSeek-R1	9.4 (1.0)	9.5 (1.1)	9.4 (0.8)	9.3 (1.0)

#### Normality and Homogeneity of Variance Tests

Shapiro–Wilk test showed that score data for all problem types conformed to normal distribution (*P*>.05). Levene test indicated homogeneity of variance for medication consultation (*P*=.12), medication education (*P*=.09), prescription review (*P*=.15), and case analysis (*P*=.10).

#### ANOVA

Across all problem types, significant differences were observed in scores among different generative AI dialogue systems (*P*<.001), with effect size η²>0.3, indicating statistically significant differences (Table 2).

**Table 2.** ANOVA results (one-way, *α*=.05).

Question type	F (*df*) value	*P* value	η² (effect size)	Significance conclusion
Medication consultation	14.3 (7, 44)	<.001	0.36	Significant differences exist
Medication education	12.8 (7, 44)	<.001	0.32	Significant differences exist
Prescription audit	16.5 (7, 44)	<.001	0.40	Significant differences exist
Case analysis and pharmaceutical care	18.1 (7, 44)	<.001	0.43	Significant differences exist

#### Multiple Comparisons (Tukey HSD)

DeepSeek-R1 demonstrated the best overall performance across all 4 task categories, with particularly significant advantages in prescription review, case analysis, and pharmaceutical care tasks. Qwen and GPT-4o showed similar performance in most tasks with no significant differences. Kimi and Doubao exhibited significant gaps (*P*<.05) in certain tasks when compared to DeepSeek-R1. ERNIE Bot consistently underperformed, with highly significant differences (*P*<.001) compared with other models.

Specifically, for medication consultation, DeepSeek-R1 was extremely significantly superior to ERNIE Bot (*P*<.001). For medication education, most comparison results were not significant, indicating minimal differences among generative AI dialogue systems in this aspect. For prescription review, ERNIE Bot performed the poorest, showing significant (*P*<.05) or extremely significant (*P*<.001) differences when compared to all other generative AI dialogue systems. For case analysis and pharmaceutical care, ERNIE Bot remained significantly weaker than most models (eg, very significant difference compared to GPT-4o, *P*<.01), while DeepSeek-R1 maintained stable performance with significant differences compared to all other models. See Table 3 and Figure 2.

**Table 3.** Pairwise comparison results of 8 generative artificial intelligence (AI) dialogue systems across 4 question types (Tukey Honestly Significant Difference) only display the groups with significant differences.

Task and comparison of significant difference groups	Mean difference (95% CI)		*P* value
Medication consultation			
ERNIE Bot-Qwen	–1.7 (–2.9 to –0.5)		<.001
ERNIE Bot-GPT-4o	–1.3 (–2.5 to –0.1)		.03
ERNIE Bot- Gemini-1.5-Pro	–1.5 (–2.7 to 0.27)		<.001
ERNIE Bot- Claude-3.5-Sonnet	–1.7 (–2.9 to –0.5)		<.001
ERNIE Bot-DeepSeek-R1	–2.1 (–3.2 to –0.8)		<.001
Doubao-DeepSeek-R1	–1.3 (–2.4 to –0.03)		.04
Kimi-DeepSeek-R1	–1.4 (–2.6 to –0.1)		.01
Prescription audit			
ERNIE Bot- Kimi	–1.3 (–2.5 to –0.1)		.04
ERNIE Bot-Qwen	–1.6 (–2.8 to –0.4)		.002
ERNIE Bot-GPT-4o	–1.4 (–2.6 to –0.19)		.01
ERNIE Bot- Gemini-1.5-Pro	–1.8 (–3.1 to –0.7)		<.001
ERNIE Bot- Claude-3.5-Sonnet	–1.8 (–3.1 to –0.7)		<.001
ERNIE Bot-DeepSeek-R1	–2.1 (–2.3 to 0.09)		<.001
Doubao-Qwen	–1.3 (–2.5 to –0.1)		.02
Doubao- Gemini-1.5-Pro	–1.6 (–2.8 to –0.4)		.001
Doubao - Claude-3.5-Sonnet	–1.6 (–2.8 to –0.4)		.002
Doubao-DeepSeek-R1	–1.9 (–3.1 to –0.68)		<.001
Case analysis and pharmaceutical care			
ERNIE Bot- Kimi	–1.2 (–2.4 to –0.03)		.04
ERNIE Bot-Qwen	–1.8 (–3.0 to –0.6)		<.001
ERNIE Bot- GPT-4o	–1.5 (–2.7 to –0.2)		.007
ERNIE Bot- Gemini-1.5-Pro	–1.3 (–2.5 to –0.13)		.02
ERNIE Bot- Claude-3.5-Sonnet	–1.6 (–2.8 to –0.4)		.003
ERNIE Bot- DeepSeek-R1	–2.6 (–3.8 to –1.4)		<.001
Doubao-DeepSeek-R1	–1.6 (–2.8 to –0.4)		.002
Kimi-DeepSeek-R1	–1.3 (–2.5 to –0.1)		.02
Gemini-1.5-Pro-DeepSeek-R1	–1.2 (–2.4 to –0.02)		.043

#### Interrater Reliability (ICC Values)

The ICC values for all 6 dimensions in medication consultation, medication education, and prescription review were >0.75, indicating good interrater reliability. Accuracy and logical coherence showed the highest consistency (ICC>0.8), while conciseness in case analysis and pharmaceutical care demonstrated the lowest consistency (ICC=0.70), suggesting considerable disagreement among raters when evaluating generative AI dialogue systems’ solutions to complex problems (Table 4). A higher ICC value indicates stronger consistency, while a lower ICC value reflects weaker consistency. The darker red in Figure 3 indicates higher consistency, while deeper blue indicates lower consistency.

**Table 4.** Intraclass correlation coefficient values by dimension (2-way random effects model).

Dimension	Medication consultation	Medication education	Prescription audit	Case analysis and pharmaceutical care
Accuracy	0.88	0.85	0.82	0.79
Preciseness	0.83	0.81	0.78	0.75
Applicability	0.80	0.79	0.76	0.72
Logicality	0.85	0.83	0.80	0.77
Conciseness	0.78	0.75	0.76	0.70
Universality	0.82	0.80	0.77	0.74

## Discussion

### Principal Findings

AI applications in health care demonstrate enormous development potential while facing numerous challenges [11]. This study, through constructing a 6-dimensional evaluation system, systematically compares the application effects and limitations of current mainstream conversational AI platforms, providing important evidence for further optimization of intelligent pharmaceutical systems.

### Key Findings

This study systematically evaluated the comprehensive performance of 8 mainstream generative AI systems across 4 core clinical pharmacy scenarios: medication consultation, medication education, prescription review, and case analysis with pharmaceutical care. The principal findings are as follows.

#### Significant Model Performance Stratification

DeepSeek-R1 demonstrated superior performance across all 4 task categories (composite score: medication consultation 9.4, SD 1.0, case analysis 9.3, SD 1.0), exhibiting particularly pronounced advantages in complex tasks such as case analysis with pharmaceutical care (*P*<.05).

Second-tier models (Qwen, GPT-4o, Claude-3.5-Sonnet, and Gemini-1.5-Pro) performed well in specific scenarios but consistently underperformed DeepSeek-R1 overall (eg, Gemini-1.5-Pro scored 9.2, SD 1.1 in prescription review vs DeepSeek-R1’s 9.4, SD 0.8).

Doubao and Kimi exhibited significant performance variability, while ERNIE Bot consistently lagged significantly across all tasks (eg, case analysis score=6.8, SD 1.5; *P*<.001 difference vs DeepSeek-R1).

#### Critical Limitations Exposed

Static knowledge bases and lack of localization: most models failed to adapt to Chinese clinical practices (eg, 90% incorrectly recommended macrolides for drug-resistant *Mycoplasma pneumoniae* infection; only DeepSeek-R1 correctly advised short-term doxycycline based on American Academy of Pediatrics [AAP] guidelines).

High-risk decision blind spots: models frequently overlooked critical contraindications (eg, only 25% recognized ethambutol is contraindicated in optic neuritis) and special regulatory requirements (eg, no model warned about the violation of prescribing diazepam beyond 7 d).

Complex reasoning deficiencies: in case analysis tasks, models struggled to integrate multidimensional information to formulate individualized care plans (eg, only Claude-3.5-Sonnet identified the diagnostic contradiction of “benign prostatic hyperplasia in a female patient”).

These limitations highlight 3 major technical bottlenecks: lagging knowledge base updates, insufficient complex decision-making reasoning, and prompt sensitivity risks (section “Technical Bottlenecks and Clinical Challenges” for details).

#### Rater Consistency Challenge

The “Conciseness” dimension in case analysis tasks exhibited the lowest interrater reliability (ICC=0.70), reflecting inconsistent assessment standards for complex problems.

Therefore, while current generative AI can serve as an auxiliary reference tool for clinical pharmacists, its error rate in high-risk decisions (eg, omission of contraindications) and lack of localization capability preclude its use as an independent basis for clinical decision-making.

#### Technical Bottlenecks and Clinical Challenges

##### Knowledge Base Limitations and Lag in Dynamic Update

This study found that all 8 generative AI dialogue systems shared a common deficiency in the areas of medication consultation and medication education: a lack of comprehensive disease assessment capabilities, making it difficult for them to provide personalized medication guidance. For instance, their guidance on the use of special devices was often inaccurate. When queried about the proper use of “Budesonide and Formoterol Inhaler,” most systems failed to accurately identify the specific type of device involved. Instead, they incorrectly described the usage instructions for other devices (eg, Salbutamol Aerosol), potentially misleading users.

Regarding the question “Can a 6-year-old child with *Mycoplasma pneumoniae* infection take doxycycline?” only DeepSeek-R1 provided a comprehensive answer incorporating the latest guidelines, “The risk of tooth discoloration from short-term doxycycline therapy (≤21 days) is extremely low, and organizations such as the AAP have relaxed relevant restrictions. Clinicians may consider using doxycycline in the following circumstances: when macrolides are resistant or ineffective; when the child’s condition is severe (eg, persistent high fever and lung consolidation); when no other safe alternative medications are available.” Other generative AI dialogue systems still only recommended macrolide antibiotics, which are unsuitable for China’s environment, where *Mycoplasma pneumoniae* has high resistance to macrolide antibacterial drugs. This is closely related to training data relying on static knowledge bases and lacking real-time, evidence-based pharmaceutical knowledge updates. DeepSeek, by leveraging publicly available open-source datasets to facilitate continuous learning, can enhance adaptability to evolving medical knowledge and scientific reasoning [12]. Nevertheless, DeepSeek-R1 also demonstrated notable limitations, including overly specialized terminology and a lack of concise expression. Particularly when responding to simple medication inquiries, the answers were excessively complex and lengthy, making it more suitable as a reference tool for health care professionals rather than for general users.

In addition, semantic ambiguity issues in Chinese contexts (as shown in the “prostate hyperplasia” misdiagnosis case) highlight the deficiencies in localized medical knowledge base construction. Furthermore, the international models (GPT-4o, Gemini-1.5-Pro, and Claude-3.5-Sonnet) exhibit biases in their understanding of culturally sensitive issues. For example, when queried about the risks of concomitant use of Chinese herbal injections and Warfarin, these models may overlook the impact of *CYP2C9* gene polymorphisms prevalent in East Asian populations. Addressing such culture-gene interactions necessitates optimization through localized training frameworks. Research indicates an increasingly evident trend of using transformer-based language models in various natural language processing models in the medical field [13], emphasizing the necessity of developing multilevel language training frameworks to adapt to professional medical environments [14].

##### Deficiencies in Complex Reasoning and Associated Ethical Risks

In prescription review, for a simple case “16-year-old male patient diagnosed with periapical abscess [15], prescribed levofloxacin tablets 0.2 g po bid, metronidazole tablets 0.6 g po tid, chlorhexidine acetate mouthwash 5 ml tid,” only ERNIE Bot, Doubao, and Kimi failed to identify that quinolone antibiotics are contraindicated in patients younger than 18 years of age, while the other 5 systems successfully identified this key safety issue.

However, for more complex prescriptions, such as “61-year-old male patient diagnosed with optic neuritis and tuberculous encephalopathy, prescribed: isoniazid 0.3 g qd, pyrazinamide 3 g biw, ethambutol tablets 0.75 g qd, rifampin capsules 0.6 g qd,” only Qwen and DeepSeek-R1 accurately identified the critical contraindication that ethambutol could exacerbate visual impairment in patients with optic neuritis.

Notably, for the prescription “40-year-old female patient diagnosed with insomnia, prescribed: diazepam tablets 10 mg po qn for 10 days” none of the 8 generative AI dialogue systems accurately identified the special management requirements for diazepam as a Class II psychotropic medication—that Class II psychotropic medications should not be prescribed for more than 7 days per prescription, and special circumstances require physician documentation. This regulatory requirement has significant importance in clinical pharmacists’ routine prescription review work.

Even more concerning, for the prescription “71-year-old female patient diagnosed with hypertension and prostatic hyperplasia, prescribed: amlodipine tablets 5 mg qd, irbesartan and hydrochlorothiazide tablets 150 mg:12.5 mg qd,” only Claude-3.5-Sonnet successfully identified the obvious error: the diagnosis was inconsistent with the patient’s gender (females do not have prostates and cannot be diagnosed with prostatic hyperplasia). The other 7 generative AI dialogue systems failed to detect this serious error. This indicates that generative AI systems still have serious hallucinations and cognitive limitations in medical reasoning.

In the case analysis and pharmaceutical care aspect, this study required generative AI dialogue systems to assume the role of clinical pharmacists and analyze drug therapy regimens based on patient information including basic demographics, reason for consultation, present illness, past medical history, medication history, family history, allergies, adverse reaction history, unhealthy habits, diagnoses, current medication records, and laboratory examination results. They were also asked to develop a pharmaceutical care plan. These tasks closely simulate the professional work of clinical pharmacists during daily rounds, requiring comprehensive and in-depth analysis and assessment of cases to formulate medication monitoring plans tailored to individual patient characteristics.

The results indicated that all 8 generative AI systems faced significant challenges in executing these complex professional tasks, struggling to simultaneously ensure both accuracy and comprehensiveness in their responses. This reflects the current limitations of AI systems in integrating diverse clinical information and making professional pharmaceutical decisions, particularly in complex clinical scenarios requiring consideration of multiple factors. This relates to AI’s lack of clinical contextual reasoning and insufficient understanding of complex instructions.

A recent research review indicates that AI has higher error rates when integrating multimodal data (such as laboratory indicators and imaging results) compared to single-modality tasks [16]. Before resolving these technical bottlenecks, we cannot avoid the issue of accountability for AI-driven decisions [17]. How legal responsibility should be defined when patients experience adverse reactions due to incorrect AI recommendations remains unclear with no explicit regulations currently issued [18]. Therefore, AI dialogue systems should be positioned as “augmented intelligence” tools, establishing “human-machine co-review” mechanisms [19], strengthening human supervision, and ensuring pharmacists retain final decision-making authority over AI outputs. The ethical risks associated with AI identified in this study strongly align with the medical AI regulatory requirements emphasized in the European Union AI Act [20].

##### Instruction Adherence and Stability Issues

This study used a single, independent query mode for evaluation, where each question was input to the model as an independent conversation. Under this mode, no instances were observed of the model significantly “forgetting” or ignoring core instructions (eg, “act as a pharmacist,” “incorporate the latest clinical guidelines,” and “develop a pharmaceutical care plan”) within a single response.

However, it is crucial to emphasize that this study did not test the stability of the model’s instruction adherence across continuous, multiturn dialogues. In real-world clinical deployment, users may engage in sustained conversations with an AI system involving multiple questions. Existing technical observations report that generative AI carries a risk of instruction drift or context forgetting during extended conversations or multiturn interactions. This could lead to subsequent responses deviating from the initially specified role or requirements, potentially compromising the reliability and safety of the answers.

Therefore, future research should design dedicated testing protocols to evaluate model instruction consistency in multiturn dialogues. Furthermore, exploring methods to enhance stability through prompt engineering optimization (eg, periodic instruction reinforcement) or model fine-tuning is warranted.

### Compound Error Risk

While the scoring criteria in this study focused on explicit errors (eg, omission of contraindications), they did not detect potentially hazardous information embedded within otherwise correct responses. For instance, an AI model correctly identified a drug-drug interaction but ambiguously advised to “monitor during use” without explicitly stating the need for immediate discontinuation. In pediatric dosing advice, a model correctly recommended a drug dose but failed to emphasize the necessity of weight-based adjustment. Such errors carry a risk of clinical misinterpretation. Therefore, future research may need to develop algorithms for detecting latent risks, such as real-time validation based on databases like the FDA Adverse Event Reporting System (FAERS).

### Application Opportunities and Optimization Pathways

#### Personalized Medication Decision Support

The DeepSeek-R1 model demonstrated good guideline adherence in pediatric doxycycline medication recommendations (incorporating the latest AAP recommendations), highlighting the potential application of AI in personalized medication decision support [21]. Relevant research confirms that AI can help formulate interventions under the guidance of predictive models by forecasting individual responses to treatment and monitoring patient progression, thereby modifying individualized treatment plans [22]. However, it should be noted that AI still lags significantly behind human experts in providing individualized treatment plans. For instance, Marcaccini et al [23] discovered that while AI-driven models demonstrate strong diagnostic accuracy and readability, further refinements are needed to improve treatment specificity and personalization. Looking forward, constructing an “AI pharmaceutical knowledge graph” [24] that correlates individual metabolic characteristics (such as CYP450 enzyme phenotypes) with pharmacokinetic data could provide dynamic dosing optimization strategies based on the latest evidence-based evidence for clinical practice.

#### Process Automation and Resource Optimization

Compared to clinical pharmacists, generative AI dialogue systems possess powerful information retrieval, data integration, and conversation capabilities [25]. Natural language processing technology can automatically extract and analyze data from large volumes of electronic medical records [26], which will greatly reduce manual time consumption, and the application of this technology is expected to significantly reduce the burden on clinical pharmacists in routine documentation such as medication history records. By communicating with users through verbal or nonverbal means, simulated pharmacist assistants have achieved improved medication adherence by providing medication education to older patients with diabetes [27], all of which enables more optimized resource allocation.

#### Medical Education and Skills Training

Generative AI dialogue systems provide new tools for clinical educational interventions and medical practice [28], bringing new dimensions of personalized learning, enhanced visualization, and simulation-based clinical training to the forefront [29]. In addition, AI-driven simulations offer realistic immersive training opportunities that prepare students for complex clinical situations and cultivate the interprofessional collaboration skills essential for modern health care [30].

#### Comparison With Previous Work

This study engages in significant dialogue with and extends existing literature in terms of both methodology and findings.

##### Deepened Evaluation Framework

Compared to the 3-dimensional evaluation frameworks proposed by other researchers, this study innovatively constructs a “six-dimensional evaluation system,” providing a more comprehensive capture of AI efficacy in pharmaceutical practice. Unlike single-model studies [8-10], this work presents the first cross-model comparison of 8 mainstream AI systems, revealing significant performance stratification (eg, DeepSeek-R1 significantly outperformed GPT-4o in Case Analysis tasks, *P*<.05).

##### Validation and Extension of Key Limitations

This study confirms the warning highlighted in the literature [15]. AI error rates remain high in complex medication decision-making (eg, a contraindication omission rate of 75% in Prescription Review tasks). Furthermore, semantic ambiguities unique to the Chinese context (eg, the gender contradiction in “benign prostatic hyperplasia”) resulted in higher error rates compared with monolingual settings, underscoring the urgency for cross-lingual training frameworks.

##### Coverage of Innovative Scenarios

In contrast to studies [3] limited to information retrieval, this research systematically validates, for the first time, AI performance across a continuous clinical decision-making chain (eg, from prescription review to pharmaceutical care plan development). This reveals deficiencies in multimodal data integration and contextual reasoning (resulting in higher error rates compared to single-task evaluations).

##### Incremental Contribution of This Study

Through its systematic evaluation across multiple models, diverse scenarios, and 6 dimensions, this study provides an empirical foundation for the localized adaptation pathways (eg, dynamic knowledge graph updating) and ethical deployment boundaries (eg, a “human-AI co-review” mechanism) of generative AI in clinical pharmacy. These findings call for the establishment of cross-lingual training frameworks and continuous evaluation systems.

### Limitations and Future Directions

#### Scope of Evaluated Scenarios

While this study focused on 4 core scenarios in clinical pharmacy practice, it did not comprehensively cover all potential application domains, such as pharmacovigilance signal mining, pharmacoeconomic evaluation, and public health emergency scenarios. Future studies should expand the scope of evaluation, for instance, by designing emergency medication test sets to assess model reliability under limited evidence.

#### Sample Size and Complexity

Although 48 questions were included, the number of Case Analysis and Pharmaceutical Care tasks was relatively low (only 8 questions). Furthermore, the complexity of the questions may still be insufficient to fully reflect the models’ capability to handle extremely complex, rare, or multisystem real-world cases. Future research should increase sample size, incorporate more diverse and higher-complexity real-world cases, and consider supplementing data using Standardized Patient Data or synthetic data generation techniques.

#### Prompt Sensitivity

Generative AI systems exhibited significant sensitivity to the wording and structure of input prompts (Prompt Sensitivity). Minor variations in question phrasing (eg, adjusting keyword order, adding or removing qualifiers) could lead to divergent responses. While this study mitigated this variability through standardized instruction templates, it did not systematically quantify the impact of this sensitivity on the results. In addition, the 3 international models (GPT-4o, Gemini-1.5-Pro, and Claude-3.5-Sonnet) may have limited comprehension of Chinese medical terminology. Future research could use joint embedding models to reduce semantic bias or use adversarial prompt testing to evaluate model robustness and optimize instruction design.

#### Model Dynamic Updates

AI models undergo rapid iteration and updates (eg, Gemini 1.5 Pro updates monthly). This study reflects the performance of specific model versions at a fixed point in time (February 20, 2025), and findings may change with subsequent model updates. There is an urgent need to establish continuous, dynamic evaluation frameworks and benchmarks to track the evolution of model performance.

#### Insufficient Visualization of Results

This study recorded and summarized the total scores for each model across the 4 task types. However, it did not calculate the average scores for each model across the 6 evaluation dimensions (spanning all 4 task types). Consequently, it cannot intuitively display each model’s relative strengths and weaknesses across dimensions or facilitate cross-model performance comparisons by dimension (eg, using radar charts). Future evaluations should incorporate these metrics.

#### Lack of Real-World Impact Assessment

The study evaluated model output quality in a controlled environment but did not assess the actual impact on pharmacist workflow efficiency, decision-making quality, or patient outcomes in real-world clinical settings. Future research should conduct prospective implementation studies or randomized controlled trials.

### Conclusions

This study conducted a systematic evaluation and comparative analysis of the application efficacy of 8 mainstream domestic and international generative AI systems across 4 core clinical pharmacy practice scenarios by constructing a 6-dimensional evaluation system. The results demonstrate that DeepSeek-R1 outperformed other models in overall performance, exhibiting particularly significant advantages in handling complex case analysis and pharmaceutical care tasks. However, all models exhibited limitations, prominently manifested in lagging knowledge base updates (eg, incorrect instructions for special devices and lack of localized recommendations), insufficient complex decision-making reasoning capabilities (eg, failure to identify critical medication contraindications and special regulatory requirements), sensitivity to prompt instructions, and overly technical terminology in outputs. Interrater reliability analysis revealed substantial disagreement in evaluating the conciseness dimension for complex tasks such as case analysis.

Based on these findings, this study concludes that while current generative AI systems demonstrate significant potential for efficiency gains and value as decision-support tools in clinical pharmacy, their responses still contain non-negligible errors and limitations, particularly at high-risk decision points. Therefore, at this stage, they should be strictly positioned as auxiliary reference tools for clinical pharmacists, not as independent bases for clinical decision-making. Future development should focus on overcoming key bottlenecks, including achieving dynamic knowledge updating and localization adaptation, enhancing reasoning capabilities in complex scenarios, improving prompt robustness and output interpretability, and establishing continuous evaluation mechanisms. Ultimately, safe, reliable, and patient-centered intelligent pharmaceutical care systems should be built through interdisciplinary collaboration integrating evidence-based medicine, ethical norms, and technological innovation.

## Supplementary material

10.2196/76128 Multimedia Appendix 1 Questions 1-48.

## Abbreviations

AAP: American Academy of Pediatrics
AI: artificial intelligence
CHA: Chinese Hospital Association
CMA: Chinese Medical Association
FAERS: FDA Adverse Event Reporting System
HSD: Honestly Significant Difference
ICC: intraclass correlation coefficient
MTM: Medication Therapy Management
