# Supplementary material for: Comparative Analysis of Generative Artificial Intelligence Systems in Solving Clinical Pharmacy Problems: Mixed Methods Study
Source: JMIR Med Inform. 2025 Jul 24;13:e76128. doi: 10.2196/76128 (PMC12288765; doi:10.2196/76128)
Supplement: Multimedia Appendix 1 [file medinform-v13-e76128-s001.doc]

**Ⅰ.Section I Medication‐Use Enquiries (20 items)**

(The questions below encompass ten key topics—indications/efficacy, administration, dosage, precautions, drug–drug interactions, storage, identification and management of adverse reactions, special-formulation handling, use in special populations, and disease prevention—two questions for each topic.)

1. Acting as a clinical pharmacist and referring to the latest clinical guidelines and evidence, please answer:
   • When should rosuvastatin be taken?
   • Must it always be taken at bedtime?
2. Acting as a clinical pharmacist and referring to the latest clinical guidelines and evidence, please answer:
   • During breastfeeding, is it permissible to take ibuprofen for a cold with fever?
3. Acting as a clinical pharmacist and referring to the latest clinical guidelines and evidence, please answer:
   • A daily dose of warfarin was missed today—what should the patient do?
4. Acting as a clinical pharmacist and referring to the latest clinical guidelines and evidence, please answer:
   • Does the insulin pen used every day have to be kept refrigerated?
5. Acting as a clinical pharmacist and referring to the latest clinical guidelines and evidence, please answer:
   • After three months of rosuvastatin therapy, the patient now has arm pain—what should be done?
6. Acting as a clinical pharmacist and referring to the latest clinical guidelines and evidence, please answer:
   • How should budesonide/formoterol inhaler be used?
   • Is long-term use acceptable?
7. Acting as a clinical pharmacist and referring to the latest clinical guidelines and evidence, please answer:
   • A patient with a cold and fever took an antipyretic three hours ago but still has a high temperature—may another dose be taken now?
8. Acting as a clinical pharmacist and referring to the latest clinical guidelines and evidence, please answer:
   • A patient with diabetes and hypertension for more than one year has been adherent to medication; current blood pressure is 140/90 mmHg and the patient feels fine—may the antihypertensives be discontinued?
9. Acting as a clinical pharmacist and referring to the latest clinical guidelines and evidence, please answer:
   • Recurrent aphthous ulcers—what medicines can be used?
   • Are antibiotics appropriate?
10. Acting as a clinical pharmacist and referring to the latest clinical guidelines and evidence, please answer:
    • How should metronidazole vaginal gel be used?
11. Acting as a clinical pharmacist and referring to the latest clinical guidelines and evidence, please answer:
    • After taking levofloxacin tablets the patient is unable to sleep—what can be done?
12. Acting as a clinical pharmacist and referring to the latest clinical guidelines and evidence, please answer:
    • The patient developed asthma while on aspirin last year. Now, due to joint pain, ibuprofen is being considered—can it be taken?
13. Acting as a clinical pharmacist and referring to the latest clinical guidelines and evidence, please answer:
    • Can nitroglycerin be used for the prevention and treatment of angina pectoris?
14. Acting as a clinical pharmacist and referring to the latest clinical guidelines and evidence, please answer:
    • A patient with Parkinson’s disease is taking levodopa. May a multivitamin be taken concurrently?
15. Acting as a clinical pharmacist and referring to the latest clinical guidelines and evidence, please answer:
    • A 69-year-old patient has a blood pressure of 140/90 mmHg—is this hypertension?
    • How should it be managed?
16. Acting as a clinical pharmacist and referring to the latest clinical guidelines and evidence, please answer:
    • The patient previously took simvastatin 40 mg; this has now been switched to rosuvastatin 5 mg. Because the milligram doses differ greatly, will lipid-lowering efficacy decline?
17. Acting as a clinical pharmacist and referring to the latest clinical guidelines and evidence, please answer:
    • The patient is allergic to penicillin—may amoxicillin capsules be used?
18. Acting as a clinical pharmacist and referring to the latest clinical guidelines and evidence, please answer:
    • For diarrhea the physician prescribed norfloxacin capsules and a live combined Bifidobacterium preparation. Can these two medicines be taken together?
19. Acting as a clinical pharmacist and referring to the latest clinical guidelines and evidence, please answer:
    • Human albumin purchased at the pharmacy must be refrigerated. The journey home takes 3–4 hours in summer. Will the product spoil due to high temperature during transit?
20. Acting as a clinical pharmacist and referring to the latest clinical guidelines and evidence, please answer:
    • Can a 6-year-old child with Mycoplasma pneumoniae infection be treated with doxycycline?

**II. Medication Counseling (10 questions)**

(Scoring criteria: clear logic and accurate content; correct identification of the diagnosis; precise recognition of drug indications, dosage & administration, and common adverse reactions; provision of appropriate medication advice in line with the therapeutic regimen.)

1. Acting as a clinical pharmacist and referring to the latest clinical guidelines and evidence, answer the following:
   A 53-year-old man has been diagnosed with coronary heart disease, hypertension, and hyperlipidemia. The physician has prescribed:
   • Aspirin enteric-coated tablets 100 mg once nightly
   • Simvastatin tablets 10 mg once nightly
   • Amlodipine besylate tablets 5 mg once daily
   • Metoprolol tartrate tablets 12.5 mg twice daily
   Please prepare medication-education material for this patient.
2. Acting as a clinical pharmacist and referring to the latest clinical guidelines and evidence, answer the following:
   A 2-year-old boy has been diagnosed with an upper-respiratory bacterial infection. The physician has prescribed:
   • Paracetamol suppository, 1 piece, rectally
   • Cefaclor dry suspension 0.125 g three times daily
   • Montelukast sodium chewable tablets 4 mg once nightly
   Please prepare medication-education material for this patient.
3. Acting as a clinical pharmacist and referring to the latest clinical guidelines and evidence, answer the following:
   A 30-year-old woman has been diagnosed with infectious diarrhea. The physician has prescribed:
   • Levofloxacin tablets 500 mg once daily
   • Bifidobacterium triple viable capsules 0.42 g twice daily
   Please prepare medication-education material for this patient.
4. Acting as a clinical pharmacist and referring to the latest clinical guidelines and evidence, answer the following:
   A 60-year-old woman has been diagnosed with atrial fibrillation. The physician has prescribed:
   • Warfarin tablets 2.5 mg once daily
   Please prepare medication-education material for this patient.
5. Acting as a clinical pharmacist and referring to the latest clinical guidelines and evidence, answer the following:
   A 56-year-old man has been diagnosed with type 2 diabetes mellitus and grade-2 hypertension (very-high-risk group). The physician has prescribed:
   • Glimepiride tablets 2 mg once daily
   • Metformin tablets 0.5 g three times daily
   • Felodipine sustained-release tablets 5 mg once daily
   Please prepare medication-education material for this patient.
6. Acting as a clinical pharmacist and referring to the latest clinical guidelines and evidence, answer the following:
   A 75-year-old man has been diagnosed with chronic obstructive pulmonary disease (COPD). The physician has prescribed:
   • Tiotropium bromide inhalation powder (device included) 18 µg per dose, once daily by inhalation
   Please prepare medication-education material for this patient.
7. Acting as a clinical pharmacist and referring to the latest clinical guidelines and evidence, answer the following:
   A 39-year-old man has been diagnosed with hyperuricemia. The physician has prescribed:
   • Benzbromarone 50 mg once daily
   • Sodium bicarbonate tablets 1 g three times daily
   Please prepare medication-education material for this patient.
8. Acting as a clinical pharmacist and referring to the latest clinical guidelines and evidence, answer the following:
   A 58-year-old man has been diagnosed with lung cancer. The physician has prescribed:
   • Fentanyl transdermal patch 4.2 mg, external use, one patch every three days
   Please prepare medication-education material for this patient.
9. Acting as a clinical pharmacist and referring to the latest clinical guidelines and evidence, answer the following:
   A 56-year-old man has been diagnosed with membranous nephropathy. The physician has prescribed:
   • Tacrolimus capsules 2 mg twice daily
   Please prepare medication-education material for this patient.
10. Acting as a clinical pharmacist and referring to the latest clinical guidelines and evidence, answer the following:
    A 78-year-old man has been diagnosed with bronchial asthma. The physician has prescribed:
    • Doxofylline tablets 0.2 g twice daily
    Please prepare medication-education material for this patient.

**III. Prescription Review (10 Questions)**

1. Acting as a pharmacist and applying current pharmacotherapeutic knowledge together with the latest clinical guidelines, determine whether the following prescription is erroneous and state your reasons.
   Patient: 50-year-old male, diagnosed with gastric ulcer, H. pylori-positive.
   Prescription: omeprazole 20 mg bid; clarithromycin 500 mg bid.
   (Scoring criteria: ability to recognize that one antibiotic is missing from the standard H. pylori triple-therapy regimen and/or that the dosing schedule is inappropriate.)
2. Acting as a pharmacist and applying current pharmacotherapeutic knowledge together with the latest clinical guidelines, determine whether the following prescription is erroneous and state your reasons.
   Patient: 50-year-old male, diagnosed with gastric ulcer, H. pylori-positive.
   Prescription: omeprazole 20 mg bid; clarithromycin 500 mg bid; amoxicillin 0.25 g bid.
   (Scoring criteria: ability to identify whether any specific drug’s dosage or administration frequency is incorrect/inappropriate.)
3. Acting as a pharmacist and applying current pharmacotherapeutic knowledge together with the latest clinical guidelines, determine whether the following prescription is erroneous and state your reasons.
   Patient: 69-year-old female, diagnosed with chronic bronchial asthma.
   Prescription: roxithromycin sustained-release capsules 300 mg qd; aminophylline 0.2 g tid.
   (Scoring criteria: ability to recognize drug–drug interactions and/or inappropriate combination therapy.)
4. Acting as a pharmacist and applying current pharmacotherapeutic knowledge together with the latest clinical guidelines, determine whether the following prescription is erroneous and state your reasons.
   Patient: 25-year-old male, diagnosed with superficial gastritis.
   Prescription: omeprazole 20 mg bid; famotidine 20 mg bid.
   (Scoring criteria: ability to identify therapeutic duplication and/or inappropriate combination therapy.)
5. Acting as a pharmacist and applying current pharmacotherapeutic knowledge together with the latest clinical guidelines, determine whether the following prescription is erroneous and state your reasons.
   Patient: 25-year-old female, diagnosed with acne.
   Prescription: cefixime dispersible tablets 200 mg bid.
   (Scoring criteria: ability to recognize inappropriate drug selection.)
6. Acting as a pharmacist and applying current pharmacotherapeutic knowledge together with the latest clinical guidelines, determine whether the following prescription is erroneous and state your reasons.
   Patient: 58-year-old female, diagnosed with angina pectoris.
   Prescription: nitroglycerin tablets 0.5 mg po prn.
   (Scoring criteria: ability to recognize an inappropriate route of administration.)
7. Acting as a pharmacist and applying current pharmacotherapeutic knowledge together with the latest clinical guidelines, determine whether the following prescription is erroneous and state your reasons.
   Patient: 61-year-old male, diagnosed with optic neuritis and tuberculous encephalopathy.
   Prescription: isoniazid 0.3 g qd; pyrazinamide 3 g biw; ethambutol 0.75 g qd; rifampicin 0.6 g qd.
   (Scoring criteria: ability to identify contraindications.)
8. Acting as a pharmacist and applying current pharmacotherapeutic knowledge together with the latest clinical guidelines, determine whether the following prescription is erroneous and state your reasons.
   Patient: 16-year-old male, diagnosed with periapical abscess.
   Prescription: levofloxacin tablets 0.2 g po bid; metronidazole tablets 0.6 g po tid; chlorhexidine acetate mouth-rinse 5 ml gargle tid.
   (Scoring criteria: ability to identify contraindications.)
9. Acting as a pharmacist and applying current pharmacotherapeutic knowledge together with the latest clinical guidelines, determine whether the following prescription is erroneous and state your reasons.
   Patient: 40-year-old female, diagnosed with insomnia.
   Prescription: diazepam tablets 10 mg po qn × 10 days.
   (Scoring criteria: ability to recognize quantity restrictions for Schedule II psychotropic drugs and/or an inappropriate treatment duration.)
10. Acting as a pharmacist and applying current pharmacotherapeutic knowledge together with the latest clinical guidelines, determine whether the following prescription is erroneous and state your reasons.
    Patient: 71-year-old female, diagnosed with hypertension and prostatic hyperplasia.
    Prescription: amlodipine tablets 5 mg qd; irbesartan/hydrochlorothiazide tablets 150 mg/12.5 mg qd.
    (Scoring criteria: ability to recognize an inappropriate clinical diagnosis.)

**IV. Case Analysis and Pharmacotherapeutic Monitoring (8 cases)**

Common chronic disease scenarios involved: coronary artery disease, hypertension, type 2 diabetes, asthma, gout.

## Case 1

As a pharmacist, please analyse the patient’s drug therapy on the basis of the following information—demographics, chief complaint, present illness, past medical history, medication history, family history, allergy history, adverse-reaction history, social history, diagnoses, current medication list and ancillary test results—and then prepare a pharmacotherapeutic monitoring plan focusing on four dimensions:

1. Indications
2. Effectiveness
3. Safety
4. Adherence

### Patient Profile

• Sex/age: Male, 76 y
• Weight: 59 kg

### Chief Complaints

1. Recent onset of constipation—requesting cause analysis and treatment advice.
2. Feels he is taking “too many” medicines—asking whether any drugs can be deprescribed.

### Present Illness

A pulmonary nodule was detected during a physical exam two weeks ago but was ignored. He later presented with back pain; CT suggested lung cancer. He was discharged after starting gefitinib (targeted therapy) and oxycodone (analgesia).

### Past Medical History

• Chronic obstructive pulmonary disease (COPD) × ≈10 years
• Benign prostatic hyperplasia (BPH) × 8 years
• Reflux oesophagitis × 0.5 year

### Prior/Current Medication History

Previously or currently used:
– Compound Methoxyphenamine Capsules
– Aminophylline Tablets
– Glycine Theophyllinate Sodium Sustained-release Tablets
– Tamsulosin Sustained-release Capsules
– Finasteride Tablets
– Rabeprazole Sodium Enteric-coated Capsules
– Tramadol/Acetaminophen Tablets
– Celecoxib Capsules
– Lactulose Oral Solution

### Family History

Non-contributory.

### Allergy History

Denies drug- or food-related allergies.

### Adverse-Reaction History

Allergy to iodixanol, manifested as rash with pruritus; relieved after symptomatic antiallergic therapy.

### Social History

Denies smoking, alcohol consumption or substance abuse.

### Diagnoses

1. Lung cancer
2. Cancer-related pain (moderate)
3. COPD
4. BPH
5. Reflux oesophagitis

### Current Medication List

• Tramadol/Acetaminophen 37.5 mg/325 mg q.i.d.
• Celecoxib 200 mg b.i.d.
• Oxycodone SR 10 mg b.i.d. (constipation noted)
• Gefitinib 250 mg q.d.
• Lactulose oral solution 15 mL b.i.d.
• Compound Methoxyphenamine 50 mg t.i.d.
• Glycine Theophyllinate Na SR 100 mg b.i.d.
• Aminophylline 0.25 g t.i.d.
• Tamsulosin SR 0.2 mg q.d.
• Finasteride 5 mg q.d.
• Rabeprazole Na EC 10 mg q.d.

### Vital Signs

BP 135/67 mmHg HR 88 bpm T 36.6 °C RR 18/min

### Laboratory Findings

Urinalysis: urobilinogen +
CBC: RBC 3.50 × 10¹²/L; Hb 103 g/L; Hct 30.8 %; Large-platelet ratio 13.2 %; PDW 8.0 %
Comprehensive biochemistry: ALT 11 U/L; AST 14 U/L; CK 41 U/L; Albumin 31.6 g/L; A/G 0.94; ALP 280 U/L; TBil 5 µmol/L; Creatinine 64 µmol/L; Urea 2.8 mmol/L; K⁺ 3.21 mmol/L; Ca²⁺ 2.02 mmol/L; TG 4.48 mmol/L; Total-C 5.28 mmol/L; LDL-C 3.12 mmol/L; HDL-C 0.95 mmol/L
Tumour markers: CEA 5.22 ng/mL; CA72-4 9.13 U/mL; PSA 12.368 ng/mL

### Imaging

1. Whole-body bone scan (MDP): Increased inorganic-salt uptake in T3-5 vertebrae and adjacent left ribs, T12, right 2nd anterior rib, right sacro-iliac joint—suggestive of multiple bone metastases.
2. Cranial spiral CT (non-contrast): Lacunar infarctions and ischaemic foci in left frontal lobe, periventricular areas and bilateral basal ganglia; senile cerebral changes.

### Case 2

As a pharmacist, please analyse this patient’s pharmacotherapy on the basis of the data below—demographic information, chief complaints, history of present illness, past history, prior medication history, family history, allergy history, adverse-reaction history, social history, diagnoses, current medication list and ancillary test results—and then draw up a pharmacotherapeutic monitoring plan addressing four dimensions:

1. Indications
2. Effectiveness
3. Safety
4. Adherence

#### Demographics

• Sex/age: Male, 70 y
• Weight: 71 kg

#### Chief Complaints

1. Believes the current drug regimen is sub-optimal and requests adjustment.
2. Has developed dysuria after taking the prescribed medicines.

#### History of Present Illness

– Persistent cough and expectoration for ≈11 years, most pronounced in the early morning; sputum usually whitish, occasionally yellow and viscous. Exacerbations precipitated by cold exposure, viral infections and smoking; symptoms worsen every winter‒spring and ease in summer, lasting about three months per year.
– Hospitalised in 2015, 2017 and 2021 for “acute exacerbation of COPD; coronary heart disease—angina; fatty liver”; improved after anti-infective, bronchodilator and symptomatic therapy.
– Two months ago, developed chest tightness and dyspnoea without obvious trigger, accompanied by worsening cough and sputum production; symptoms triggered by brisk walking or even slow walking over 50 m, partially relieved by treatment (anti-infective, nebulisation, oxygen) at a community hospital.
– Eleven days ago caught a cold; chest tightness and dyspnoea worsened, with rhinorrhoea and occasional dizziness. The same 50 m exertion precipitates chest tightness, shortness of breath and wheeze, relieved by rest. Intermittent cough has intensified; sputum whitish, quantity increased; marked fatigue.

#### Past Medical History

• Coronary heart disease, stable angina × > 2 years
• Chronic obstructive pulmonary disease (COPD)

#### Prior Medication History

Aspirin enteric-coated tablets, atorvastatin, isosorbide-5-mononitrate, budesonide/formoterol pressurised inhaler.

#### Family History

Non-contributory.

#### Allergy History

Denies drug or food allergies.

#### Adverse-Reaction History

None reported.

#### Social History

• Smoking: 20 cigarettes/day for 40 years; quit 6 months ago.
• Alcohol: occasional, ≈ 100 mL/time.
• No other substance misuse.

#### Diagnoses

1. Acute exacerbation of COPD with infection; hypoxaemia
2. Atherosclerotic coronary heart disease

#### Current Medication List

• Budesonide/Formoterol dry-powder inhaler 160 µg/4.5 µg b.i.d.
• Tiotropium dry-powder inhaler 18 µg q.d.
• Montelukast chewable tablet 5 mg q.h.s.
• Cineole/Pinene (Eucalyptus-Myrtus) enteric-coated capsule 0.12 g t.i.d.
• Isosorbide-5-mononitrate sustained-release capsule 40 mg q.h.s.
• Aspirin enteric-coated tablet 100 mg q.h.s.
• Atorvastatin calcium tablet 20 mg q.h.s.

#### Ancillary Test Results

Vital signs: BP 148/95 mmHg, HR 88 bpm, T 36.2 °C, RR 20/min

Laboratory data

1. CBC: WBC 4.71 × 10⁹/L; Neutrophils 75.6 %; Hb 154 g/L; PLT 260 × 10⁹/L
2. C-reactive protein: 0.57 mg/L
3. HbA₁c: 5.5 %
4. Chemistry: normal hepatic panel; creatinine 65 µmol/L; uric acid 341 µmol/L

Imaging
Chest CT: features of COPD, pulmonary hypertension, right upper-lobe mass, and multiple pulmonary nodules in both lungs.

**Case 3**

As a pharmacist, please analyse this patient’s pharmacotherapy on the basis of the following information—demographic data, chief complaints, history of present illness, past medical history, previous medication history, family history, allergy history, adverse-reaction history, social history, diagnoses, current medication list and ancillary test results—and then draw up a pharmacotherapeutic monitoring plan focusing on four dimensions:

1. Indications
2. Effectiveness
3. Safety
4. Adherence

**Demographics**

• Sex/age: Male, 62 y
• Weight: 70 kg

**Chief Complaints**

1. Feels the current drug therapy is sub-optimal; marked chest tightness and dyspnoea—requests regimen adjustment.
2. Poor blood-pressure control recently—peaks up to 168/102 mmHg.

**History of Present Illness**

– Chest tightness and shortness of breath for > 1 year.
– Diagnosed with “heart failure” at another hospital 1 month ago; started perindopril and spironolactone.
– Four days ago, after emotional agitation, experienced paroxysmal chest tightness (duration undocumented) accompanied by bilateral leg weakness; no chest pain, occasional palpitations; no nocturnal dyspnoea, cough, sputum, chills, fever, headache, dizziness, paralysis or aphasia. Symptoms have recurred without treatment, prompting presentation for systematic evaluation.
– During the course, blood pressure rose to 178/112 mmHg. Appetite poor; sleep acceptable; bowel and bladder normal.

**Past Medical History**

• Type 2 diabetes mellitus × 15 y
• Lacunar cerebral infarction × 3 y
• Coronary heart disease × 5 y; percutaneous coronary stenting 1 y 3 mo ago
• Hypertension × 10 y
• Heart failure × 6 mo

**Previous Medication History**

Aspirin enteric-coated tablets, clopidogrel bisulfate, isosorbide 5-mononitrate tablets, perindopril tablets, spironolactone tablets, acarbose tablets, metformin hydrochloride tablets, insulin glargine, insulin aspart.

**Family History**

Unremarkable.

**Allergy History**

Denies drug or food allergies.

**Adverse-Reaction History**

None reported.

**Social History**

• Smoking: 20 cigarettes/day for 45 years; not quit.
• Alcohol: none.
• No other substance misuse.

**Diagnoses**

1. Type 2 diabetes mellitus
2. Lacunar cerebral infarction
3. Coronary heart disease
4. Post-coronary stent implantation
5. Hypertension
6. Heart failure

**Current Medication List**

• Metformin hydrochloride tablets 0.5 g t.i.d.
• Insulin lispro protamine/insulin lispro (insulin lispro Mix 25) 20 IU t.i.d.
• Insulin glargine 22 IU q.h.s.
• Isosorbide 5-mononitrate tablets 10 mg t.i.d.
• Aspirin enteric-coated tablets 100 mg q.h.s.
• Clopidogrel bisulfate tablets 75 mg q.d.
• Perindopril tablets 4 mg q.d.
• Spironolactone tablets 20 mg q.d.

**Ancillary Test Results**

Vital signs
BP 154/98 mmHg HR 84 bpm T 36.5 °C RR 19/min

Laboratory data

1. CBC: Lymphocytes 10.72 %; Neutrophils 81.81 %
2. HbA₁c: 6.8 %
3. Comprehensive biochemistry: hepatic panel normal; creatinine 90.8 µmol/L; uric acid 526.6 µmol/L; total cholesterol 5.92 mmol/L; triglycerides 3.85 mmol/L; HDL-C 0.81 mmol/L; LDL-C 3.42 mmol/L; Na⁺ 137 mmol/L; K⁺ 4.6 mmol/L

Urinalysis & sediment: within normal limits

Imaging

1. Carotid ultrasound: bilateral atherosclerotic plaques
2. Lower-limb arterial ultrasound: bilateral atherosclerotic plaques
3. Chest CT: interstitial changes in right lung; bilateral pleural thickening with calcification
4. Ambulatory BP monitoring: fluctuates 154–120 / 78–101 mmHg
5. Echocardiography: enlarged left heart; regional LV wall-motion abnormalities; aortic sclerosis with valvular calcification and mild regurgitation; mild mitral and tricuspid regurgitation; markedly reduced LV systolic function; LVEF 38 %

**Case 4**

As a pharmacist, please analyse this patient’s pharmacotherapy on the basis of the following information—demographic data, chief complaints, history of present illness, past medical history, previous medication history, family history, allergy history, adverse-reaction history, social history, diagnoses, current medication list and ancillary test results—and then draw up a pharmacotherapeutic monitoring plan focusing on four dimensions:

1. Indications
2. Effectiveness
3. Safety
4. Adherence

**Demographics**
• Male, 69 years old, 61 kg

**Reason for Admission (Chief Complaint)**

• Acute exacerbation of chronic obstructive pulmonary disease (AECOPD).

**Present Illness History**

The patient developed cough and sputum production 4 years ago without obvious trigger. No wheezing was noted and no formal treatment was sought.
• Oct 2021: Treated with oral corticosteroids for an acute exacerbation.
• Jan 2022: New-onset wheezing with dyspnea, nocturnal orthopnea relieved by sitting upright. Pulmonary function testing at our hospital confirmed COPD.
• Current episode: Re-admitted via the outpatient department with the diagnosis “AECOPD” for follow-up and management.

**Past Medical History**

• Type 2 diabetes mellitus (T2DM) – 2 years.
• Essential hypertension – 2 years.

**Previous Medication History**

• Tiotropium bromide inhalation powder
• Alpha-pinene/limonene enteric-coated soft capsules (ELOM-080)
• Prednisone
• Metoprolol succinate extended-release tablets
• Metformin hydrochloride tablets
• Dapagliflozin tablets

**Family History**

• Non-contributory.

**Allergy History**

• Denies any known drug or food allergies.

**Adverse Drug Reaction History**

• None reported.

**Social History / Substance Use**

• Smoking: > 40 years, approximately 800 cigarettes per year; ceased May 2021.
• Alcohol: > 40 years, ~250 mL of strong liquor daily; ceased May 2021.

**Current Diagnoses**

1. Chronic obstructive pulmonary disease, GOLD group B with acute exacerbation; pulmonary function grade IV
2. Essential hypertension, grade 1 (high-risk)
3. Type 2 diabetes mellitus

**Current Medication List**

• Metoprolol succinate ER 47.5 mg orally once daily
• Metformin SR 1 g orally twice daily
• Dapagliflozin 10 mg orally every evening
• Budesonide/Glycopyrronium/Formoterol fumarate inhalation aerosol 344 µg twice daily
• Compound Methoxyphenamine 12.5 mg orally three times daily

**Vital Signs**

• Blood pressure 120/80 mmHg
• Heart rate 80 beats/min
• Temperature 36.7 °C
• Respiratory rate 18 breaths/min

**Laboratory Data**

• Urinalysis: within normal limits

1. Complete blood count
   – WBC 6.02 × 10⁹/L
   – Neutrophils 72.3 %
   – Lymphocytes 16.9 %
   – Eosinophils 0.08 × 10⁹/L
   – Hemoglobin 144 g/L
   – Platelets 174 × 10⁹/L
2. HbA1c 5.2 %
3. Liver & renal function
   – Complement C1q 244.0 mg/L ↑
   – Total protein 59 g/L ↓
   – Albumin 36 g/L ↓
   – ALT 56 U/L ↑
   – GGT 135 U/L ↑
   – Calcium 2.07 mmol/L ↓
   – Total cholesterol 5.30 mmol/L ↑
   – LDL-C 3.45 mmol/L ↑
   – Triglycerides 1.21 mmol/L
   – HDL-C 1.41 mmol/L
4. Pulmonary function
   – FEV₁/FVC 53 % (consistent with COPD)
   – FEV₁ 27 % predicted (very severe)
   – Bronchodilator reversibility: ΔFEV₁ 70 mL, 17 % (negative)
   – DLCO 94 % predicted (normal diffusion)

**Imaging**

1. Chest CT: Multiple small pulmonary nodules in both lungs.
2. Echocardiography: Normal chamber sizes; trivial tricuspid regurgitation; decreased left-ventricular compliance; normal left-ventricular systolic function.

**Case 5**

As a pharmacist, please analyse this patient’s pharmacotherapy on the basis of the following information—demographic data, chief complaints, history of present illness, past medical history, previous medication history, family history, allergy history, adverse-reaction history, social history, diagnoses, current medication list and ancillary test results—and then draw up a pharmacotherapeutic monitoring plan focusing on four dimensions:

1. Indications
2. Effectiveness
3. Safety
4. Adherence

**Demographics**
• Female, 67 years old, 54 kg

**Presenting complaints**

1. The patient feels that the current pharmacotherapy is sub-optimal and wishes to have her regimen adjusted.
2. Recent deterioration of glycaemic control: fasting plasma glucose (FPG) ≈ 8 mmol/L, 2-h post-prandial glucose ≈ 13 mmol/L; progressive paraesthesia of both upper limbs over the past month.

**History of present illness**
Five years ago the patient presented with polydipsia, polyuria, polyphagia and a 3 kg weight loss within one month. FPG was 12.8 mmol/L and HbA1c 7.2 %; she was diagnosed with type 2 diabetes mellitus (T2DM) and advised lifestyle modification plus metformin. She discontinued medication on her own and did not attend regular follow-up.
Since onset: diet and sleep acceptable; cold-induced numbness of both upper limbs; urgency of urination without dysuria or burning; alternating constipation and diarrhoea; body weight stable in recent years.
At a local outpatient clinic she was prescribed:
• Metformin 1 g PO bid
• Pioglitazone 15 mg PO qd
• Linagliptin 5 mg PO qd
• Insulin glargine 10 IU SC at bedtime

She reports good adherence but inadequate glycaemic control (FPG ≈ 8 mmol/L; 2-h PPG ≈ 13 mmol/L) and worsening upper-limb numbness over the past month. Today she attended our clinic; random glucose was 12.1 mmol/L and HbA1c 8.4 %. She was admitted for further evaluation and treatment.

**Past medical history**
• Type 2 diabetes mellitus, 5 + years
• Hypertension, 10 + years
• Dyslipidaemia, 5 + years
• Osteoporosis, 2 + years

**Previous medication history**
Metformin, acarbose, glimepiride, pioglitazone, linagliptin, insulin glargine, nifedipine controlled-release, atorvastatin, zoledronic acid.

**Family history**
Unremarkable.

**Allergy history**
Denies drug or food allergies.

**Adverse reaction history**
None reported.

**Social history**
No smoking or alcohol consumption.

**Current diagnoses**

1. Type 2 diabetes mellitus
2. Diabetic peripheral neuropathy
3. Hypertension, grade 2, very high risk
4. Dyslipidaemia
5. Osteoporosis

**Current medication list**
• Metformin 1 g PO bid
• Pioglitazone 15 mg PO qd
• Linagliptin 5 mg PO qd
• Insulin glargine 10 IU SC HS
• Nifedipine controlled-release 30 mg PO qd
• Atorvastatin 10 mg PO HS
• Calcitriol 0.25 µg PO HS
• Calcium carbonate + vitamin D₃ 600 mg PO bid

**Vital signs**
BP 147/92 mmHg, HR 72 bpm, T 36.4 °C, RR 18/min

**Laboratory data**
Urinalysis: glucose (+), ketone (−), occult blood (−)
Complete blood count: within normal limits
HbA1c: 8.40 %
Comprehensive metabolic panel:
– Liver function: normal
– Creatinine 81 µmol/L; eGFR 65.03 mL/(min·1.73 m²)
– Uric acid 463 µmol/L
– TC 2.48 mmol/L, TG 1.69 mmol/L, HDL-C 0.83 mmol/L, LDL-C 1.71 mmol/L
– Na⁺ 137 mmol/L, K⁺ 4.25 mmol/L

Capillary glucose profile:
FPG 7.1 mmol/L; 2-h post-breakfast 12.4 mmol/L; 2-h post-lunch 12.7 mmol/L; 2-h post-dinner 13.8 mmol/L; bedtime 7.4 mmol/L

Urinary renal markers:
Micro-albumin 34.58 mg/L; α₁-microglobulin 13.2 mg/L; transferrin 2.9 mg/L; UACR 65.53 mg/g

**Imaging / instrumental tests**

1. Carotid & vertebral artery duplex: bilateral atherosclerosis with multiple plaques; luminal stenosis at the origin of the left external carotid artery.
2. Lower-extremity arterial duplex: bilateral atherosclerosis with multiple plaques.
3. Fundoscopy: moderate to severe non-proliferative diabetic retinopathy, both eyes.
4. Ambulatory BP: 145–155/85–100 mmHg.
5. Echocardiography: bi-atrial enlargement, mild left-ventricular hypertrophy, impaired LV compliance.
6. DXA: T-score −3.2 at left distal radius, −4.0 at lumbar spine; diagnosis: osteoporosis.

**Case 6**

As a pharmacist, please analyse this patient’s pharmacotherapy on the basis of the following information—demographic data, chief complaints, history of present illness, past medical history, previous medication history, family history, allergy history, adverse-reaction history, social history, diagnoses, current medication list and ancillary test results—and then draw up a pharmacotherapeutic monitoring plan focusing on four dimensions:

1. Indications
2. Effectiveness
3. Safety
4. Adherence

**Basic Information**
• Patient: Male, 77 years old, 66 kg

**Reason for Visit**

1. The patient feels the current drug regimen is sub-optimal and wishes to adjust it.
2. Recurrent cough and sputum production with wheezing for 11 years, worsened for >20 days.

**History of Present Illness**
Eleven years ago, after catching cold, the patient developed cough and expectoration and was diagnosed at our hospital with “chronic obstructive pulmonary disease” (COPD). Symptoms were relieved with medication, and he has since attended our outpatient clinic regularly, with fair control of cough and dyspnea. During the past year, cough, sputum, and wheezing have relapsed frequently but were not properly managed. Twenty days ago, after another cold, cough and sputum became aggravated. Current symptoms: cough, yellow sputum, itchy throat, dry mouth; no chest tightness or wheeze; slight fatigue; normal appetite; average sleep; loose stools 2–3 times/day; concentrated urine.

**Past Medical History**
• Generally poor health
• Radical prostatectomy for prostate cancer in 2013
• Hematuria for 30 years
• Cerebral hypoperfusion for 2 years
• Impaired glucose tolerance for 6 months
• Anemia for 1 year

**Previous Medication History**
Bicalutamide tablets, goserelin sustained-release implant, folic acid tablets, vitamin B12 tablets, Yixuesheng (Chinese patent medicine), etc.

**Family History**
Non-contributory.

**Allergy History**
Denies drug or food allergies.

**Adverse Reaction History**
None reported.

**Social History / Habits**
Denies smoking and alcohol consumption.

**Diagnoses**

1. Chronic obstructive pulmonary disease (COPD)
2. Pulmonary infection
3. Bronchiectasis
4. Post-operative prostate cancer
5. Impaired glucose tolerance
6. Anemia
7. Hematuria
8. Thyroid nodule
9. Cerebral atrophy

**Current Medication Record**
• Cefoperazone/sulbactam injection 3 g IV every 8 h
• Doxofylline injection 0.3 g IV once daily
• Tiotropium bromide inhalation powder 18 µg once daily
• Bailing capsules (Cordyceps sinensis preparation) 4 capsules three times daily
• Montelukast sodium tablets 10 mg once nightly

**Auxiliary Examinations**

Vital Signs
BP 120/76 mmHg, HR 78 bpm, Temp 36.7 °C, RR 18 /min.

Urinalysis
Occult blood (BLD) 2+, RBC 77.4/µL.

Laboratory Tests

1. Complete blood count: WBC 11.2 × 10⁹/L; Neutrophils 84.9 %; Lymphocytes 44.7 %; RBC 4.03 × 10¹²/L; Hemoglobin 125 g/L; Platelets 231 × 10⁹/L.
2. HbA1c: 6.0 %.
3. Comprehensive metabolic panel: Direct bilirubin 5.6 µmol/L; Total cholesterol 2.50 mmol/L; LDL-C 1.40 mmol/L; ApoB 0.40 g/L.

Pulmonary Function

1. Overall ventilation normal; mild obstructive ventilatory defect; marked small-airway obstruction.
2. Mildly reduced diffusing capacity.
3. Bronchodilator test negative.

Fractional Exhaled Nitric Oxide
20 ppb, indicating non-eosinophilic airway inflammation.

Imaging

1. Color Doppler echocardiography: aortic valve degeneration with mild regurgitation; mild regurgitation of mitral, tricuspid and pulmonary valves; impaired left-ventricular relaxation; bilateral common carotid intima–media thickening; right subclavian artery plaque; bilateral thyroid mixed nodules; thyroid follicular formation; rough gallbladder wall.
2. Cranial MRI: cerebral white-matter demyelination; cerebral atrophy; bilateral ethmoid and frontal sinusitis; bilateral inferior turbinate hypertrophy; deviated nasal septum; no significant abnormalities on SWI or MRA.
3. Chest CT: bilateral old lesions; focal pulmonary emphysema and bullae; partial bronchiectasis; calcification of aortic wall and coronary arteries; interposed colon.

**Case 7**

As a pharmacist, please analyse this patient’s pharmacotherapy on the basis of the following information—demographic data, chief complaints, history of present illness, past medical history, previous medication history, family history, allergy history, adverse-reaction history, social history, diagnoses, current medication list and ancillary test results—and then draw up a pharmacotherapeutic monitoring plan focusing on four dimensions:

1. Indications
2. Effectiveness
3. Safety
4. Adherence

**Demographics**

Sex / Age / Weight: Female, 67 years, 65 kg

**Reason for Visit**

The patient feels that she is currently taking too many medications and wishes to simplify her regimen.

**Present Illness**

Eleven years ago the patient was hospitalized for cerebral infarction and improved after treatment. Since discharge she has experienced intermittent dizziness without an obvious pattern. During episodes there is no vertigo, loss of consciousness, transient blindness, syncope, nausea or vomiting. Symptoms remit spontaneously after rest. No decline in spatial or personal orientation, no bradykinesia, and no impaired judgement are reported. Residual left-sided motor weakness and dysarthria are present. She occasionally develops chest tightness and dyspnoea, mostly post-prandially; there is no regurgitation, heartburn, precordial pain or diaphoresis. Symptoms usually resolve after taking Compound Danshen Dripping Pills and resting. No cough, sputum production or fever. She has required multiple admissions for recurrent cerebral infarction. Since this episode she reports low spirits; appetite and sleep are acceptable; bowel and urinary habits are normal; body weight is stable.

**Past Medical History**

1. Cerebral infarction × 11 years, with residual left-sided weakness
2. Coronary heart disease × 3 years
3. Hypertension × 20 years; peak BP 210/105 mmHg; usually controlled with metoprolol succinate
4. Type 2 diabetes mellitus × 5 years; controlled with acarbose and biphasic insulin aspart 30
5. Hypothyroidism × 1 month
6. Paroxysmal atrial fibrillation × 1 year
7. Severe osteoporosis × 5 years
8. Status post left foot fracture surgery
9. History of cervical vascular stent implantation

**Previous Medication History**

Acarbose, rivaroxaban, rosuvastatin calcium, metoprolol succinate ER, propafenone, isosorbide mononitrate ER, Compound Danshen Dripping Pills, Ginkgo biloba tablets, levothyroxine sodium, ursodeoxycholic acid, bicyclol, citicoline sodium, flunarizine hydrochloride, biphasic insulin aspart 30.

**Family History**

Non-contributory.

**Allergies**

Denies drug or food allergies.

**Adverse Drug Reactions**

None reported.

**Social History**

Denies smoking and alcohol use.

**Diagnoses**

1. Ischaemic cerebrovascular disease
2. Coronary artery disease
3. Grade 3 hypertension (very high risk)
4. Type 2 diabetes mellitus
5. Hypothyroidism
6. Post-operative status, left foot fracture
7. Paroxysmal atrial fibrillation
8. Severe osteoporosis
9. Post-cervical vascular stent implantation state

**Current Medication List**

- Acarbose 50 mg PO tid
- Biphasic insulin aspart 30 12 IU SC bid
- Rivaroxaban 20 mg PO qd
- Rosuvastatin calcium 10 mg PO qhs
- Metoprolol succinate ER 23.75 mg PO qd
- Propafenone 150 mg PO tid
- Isosorbide mononitrate ER 60 mg PO qd
- Compound Danshen Dripping Pills 270 mg PO bid
- Ginkgo biloba tablets 40 mg PO tid
- Levothyroxine sodium 25 µg PO qd
- Ursodeoxycholic acid 250 mg PO bid
- Bicyclol 25 mg PO tid
- Citicoline sodium 0.2 g PO tid
- Flunarizine hydrochloride 5 mg PO qd

**Vital Signs**

Blood pressure 146/88 mmHg
Heart rate 78 bpm
Temperature 36.5 °C
Respiratory rate 18 breaths/min

**Laboratory Data**

Urinalysis: nitrite +, leukocyte esterase +++

1. Complete blood count
   • WBC 3.49 × 10⁹/L
   • RBC 4.25 × 10¹²/L
   • Hb 127 g/L
   • PLT 233 × 10⁹/L
2. HbA1c: 6.3 %
3. Comprehensive metabolic panel
   • Total cholesterol 3.81 mmol/L
   • Triglycerides 1.14 mmol/L
   • LDL-C 2.11 mmol/L
   • Glucose 5.0 mmol/L
   • Creatinine (enzymatic) 68 µmol/L
   • AST 17 U/L
   • ALT 6 U/L
   • Albumin 33 g/L
   • Potassium 4.0 mmol/L
4. Capillary glucose profile
   • Fasting 5.2 mmol/L
   • 2 h post-breakfast 8.7 mmol/L
   • 2 h post-lunch 6.5 mmol/L
   • 2 h post-dinner 10.1 mmol/L
5. Thyroid function
   • FT3 3.36 pmol/L
   • FT4 8.66 pmol/L
   • TSH (ultrasensitive) 27.443 µIU/mL
6. Immunology / Cardiac markers
   • Plasma aldosterone 8.4 ng/dL
   • Plasma renin 1.6 µIU/mL
   • NT-proBNP 135.5 pg/mL
   • CK 41 U/L
   • CK-MB 8 U/L
   • Troponin T 7.20 ng/L
7. Coagulation profile, tumour markers, stool routine: no significant abnormalities.

**Imaging Studies**

1. Cranial CT: Bilateral basal ganglia encephalomalacia, leukoaraiosis, cerebral atrophy. MRI recommended if necessary.
2. 24-h Holter: Sinus rhythm; borderline, diffuse T-wave abnormalities.
3. Echocardiography: Mild regurgitation of mitral, tricuspid and aortic valves; left-ventricular diastolic dysfunction.
4. Carotid Doppler: Post-stent status at right common-carotid bifurcation/ICA origin with patent flow; diffuse intima–media thickening and multiple plaques in bilateral carotid, vertebral and subclavian origins; < 50 % stenosis at proximal right subclavian artery.
5. Chest CT: Increased interstitial markings and changes, suggestive of interstitial inflammation; prominent cardiac silhouette; atherosclerosis; bilateral pleural thickening.
6. Abdominal ultrasound: Fatty liver; gallbladder, pancreas, spleen and kidneys unremarkable.

**Case 8**

As a pharmacist, please analyse this patient’s pharmacotherapy on the basis of the following information—demographic data, chief complaints, history of present illness, past medical history, previous medication history, family history, allergy history, adverse-reaction history, social history, diagnoses, current medication list and ancillary test results—and then draw up a pharmacotherapeutic monitoring plan focusing on four dimensions:

1. Indications
2. Effectiveness
3. Safety
4. Adherence

**Demographic data**

• Sex: Male
• Age: 60 years
• Weight: 80 kg

**Reason for consultation**

1. Pronounced fluctuation of blood glucose—how should antidiabetic therapy be modified to reach glycaemic targets?
2. Blood-pressure variability—how should antihypertensive therapy be modified to reach target BP?
3. Blurred vision—how can this be improved?
4. Fatigue and aversion toward long-term insulin injections—how can treatment adherence be enhanced?

**History of present illness**

Nineteen years ago the patient experienced unexplained weight loss of 3 kg within one month, accompanied by blurred vision and foamy urine, but without polydipsia, polyuria, polyphagia or palpitations. Fasting venous plasma glucose was 17 mmol/L; OGTT and an insulin-release test (values unavailable) confirmed type 2 diabetes mellitus (T2DM). He was started on metformin 0.5 g tid plus repaglinide 1 mg tid, achieving fasting glucose ≈ 8 mmol/L and post-prandial ≈ 10 mmol/L; foamy urine resolved, vision unchanged.
Ten years ago he self-increased repaglinide to 2 mg tid; glycaemic control remained the same.
Eight years ago, during admission to our department, duplex ultrasonography of cervical and lower-limb arteries demonstrated diabetic peripheral vascular disease; therapy was switched to pre-meal insulin aspart and bedtime insulin detemir. Glycaemic control remained unchanged.
Seven years ago, therapy was adjusted to metformin 500 mg tid + insulin aspart 10-8-8 U pre-meals + insulin glargine 8 U at bedtime.
During the past year he self-adjusted to metformin 500 mg tid + insulin aspart 10-10-10 U + insulin glargine 10 U HS, with fasting glucose 9–11 mmol/L and post-prandial 10–13 mmol/L.
He is now admitted for further management of “T2DM with diabetic peripheral vascular disease”. Over the past month he notes blurred vision; appetite, sleep and weight are stable; bowel and bladder habits normal.

**Past medical history**

• T2DM with peripheral vascular disease
• Hypertension diagnosed 3 years ago, peak 150/90 mmHg; on regular medication, usually 110–120/80–85 mmHg
• Myocardial ischaemia diagnosed 12 years ago

**Previous drug history**

Antidiabetics:
• Metformin 0.5 g po tid
• Repaglinide 1–2 mg po tid
• Insulin aspart 10/8 U sc tid
• Insulin detemir (dose unknown) sc HS
• Insulin glargine 10 U sc HS

Other drugs:
• Valsartan 80 mg po qd
• Fluvastatin 80 mg po HS
• Aspirin EC 0.1 g po HS
• Candesartan cilexetil 4 mg po qd
• Coenzyme Q10 10 mg po tid

**Family history**

Non-contributory.

**Allergy history**

Allergic to sulfonamides; no food allergies.

**Adverse-reaction history**

None reported.

**Social history**

Denies smoking and alcohol consumption.

**Current diagnoses**

1. Type 2 diabetes mellitus
2. Diabetic peripheral vascular disease
3. Grade 1 hypertension (very-high risk)
4. Decreased bone mass
5. Hepatic steatosis
6. Benign prostatic hyperplasia

**Current medication list**

• Metformin 0.5 g po tid
• Insulin aspart 10 U sc tid
• Insulin glargine 10 U sc HS
• Fluvastatin 40 mg po HS
• Valsartan 80 mg po qd
• Aspirin EC 100 mg po qd
• Coenzyme Q10 10 mg po tid
• Calcium dobesilate 0.5 g po bid
• Thioctic acid (α-lipoic acid) 600 mg iv qd

**Vital signs**

BP 147/87 mmHg; HR 71 bpm; T 36 °C; RR 18/min

**Capillary blood-glucose profile**

13 May PP lunch 6.6 mmol/L; PP dinner 7.4; bedtime 5.4
14 May FPG 5.14; PP dinner 2.9; bedtime 5.4
15 May FPG 5.3
16 May FPG 5.2; PP breakfast 10.2; PP dinner 5.7; bedtime 5.9
17 May FPG 6.2; PP lunch 8.4; PP dinner 9.1; bedtime 10.3
18 May FPG 7.5; PP lunch 9.7; PP dinner 8.0; bedtime 8.8

**Laboratory data**

• HbA₁c 6.8 % ↑
• Bone turnover: N-MID osteocalcin 9.4 ng/mL ↓; 25-OH-Vit D₃ 9.7 ng/mL ↓
• Lipids: TG 2.09 mmol/L ↑; HDL-C 0.87 mmol/L ↓; LDL-C 1.71 mmol/L ↓
• Liver enzymes: AST 15 U/L, ALT 12 U/L
• Renal: Scr 53 µmol/L ↓
• Thyroid: TSH 2.01 µIU/mL; FT3 4.06 pmol/L; FT4 15.1 pmol/L; T3 1.42 nmol/L; T4 96.3 nmol/L
• Urinalysis: ketone −, glucose −
• 24 h urinary albumin 27.4 mg/24 h ↑; ACR 22.66 mg/g

**Imaging**

1. Duplex US lower limbs: atherosclerotic plaque in left common femoral artery
2. Cervical vessels: focal IMT thickening in right subclavian and left carotid bulb
3. Echocardiogram: mild IVS hypertrophy; impaired LV relaxation
4. Abdominal US: mild fatty liver, gall-bladder polyp, BPH
5. Bone mineral density: low bone mass
